# The Anti-Stress Effect of *Mentha arvensis* in Immobilized Rats

**DOI:** 10.3390/ijms19020355

**Published:** 2018-01-25

**Authors:** Weishun Tian, Md Rashedunnabi Akanda, Anowarul Islam, Hae-Dong Yang, Sang-Cheon Lee, Jeong-Ho Lee, Sang-Ki Kim, Yu-Jin Choi, So-Yeon Im, Byung-Yong Park

**Affiliations:** 1 College of Veterinary Medicine and Bio-Safety Research Institute, Chonbuk National University, Iksan 54596, Korea; tianws0502@126.com (W.T.); rashed.mvd@gmail.com (M.R.A.); anowarul.vet@gmail.com (A.I.); 2 Department of Pharmacology and Toxicology, Sylhet Agricultural University, Sylhet 3100, Bangladesh; 3 Imsil Cheese Livestock Cooperative Association, 275 Galma-ri, Imsil-eup, Imsil-gun, Jeollabuk-do 55924, Korea; yhd1127@nonghyup.com (H.-D.Y.); korwor@nonghyup.com (S.-K.K.); 4 Imsil Cheese & Food Research Institute, 50 Doin 2-gil, Seongsu-myeon, Imsil-gun, Jeollabuk-do 55918, Korea; moiselee@icf.re.kr (S.-C.L.); samdc@nate.com (Y.-J.C.); 5 Sunchang Research Institute of Health and Longevity, 427-128 Indok-ro, Ingye-myeon, Sunchang-gun, Jeollabuk-do 56015, Korea; wooju0717@hanmail.net (J.-H.L.); soyoun5005@hanmail.net (S.-Y.I.)

**Keywords:** *Mentha arvensis*, immobilized-stress, hormones, MAPK/COX-2

## Abstract

Stress can lead to inflammation, accelerated aging, and some chronic diseases condition. *Mentha arvensis* (MA) is a traditional medicine having antioxidant and anti-inflammatory activities. The present study investigated the anti-stress role of MA and fermented MA (FMA) extract in immobilized rats. We studied the lipopolysaccharide (LPS)-induced inflammation in RAW 264.7 cells and rats were immobilized for 2 h per day for 14 days using a restraining cage. MA (100 mg/kg) and FMA (100 mg/kg) were orally administered to rats 1 h prior to immobilization. Using high-performance liquid chromatography (HPLC) analysis, we determined the rosmarinic acid content of MA and FMA. The generation of malondialdehyde (MDA) and nitric oxide (NO) in RAW 246.7 cells were suppressed by both MA and FMA. In rats, MA and FMA notably improved the body weight, daily food intake, and duodenum histology. MDA and NO level were gradually decreased by MA and FMA treatment. MA and FMA significantly controlled the stress-related hormones by decreasing corticosterone and β-endorphin and increasing serotonin level. Moreover, protein expression levels of mitogen activated protein kinases (MAPK) and cyclooxygenase-2 (COX-2) were markedly downregulated by MA and FMA. Taken together, MA and FMA could ameliorate immobilized-stress by reducing oxidative stress, regulating stress-related hormones, and MAPK/COX-2 signaling pathways in rats. Particularly, FMA has shown greater anti-stress activities than MA.

## 1. Introduction

Stress has been a significant impact on human health globally, causing several psychosomatic disorders [1] and pathological conditions, like coronary artery diseases, cancer, and diabetes mellitus, [2] and accompanying body-weight loss. The neuroendocrine damages in response to frequent stresses are managed by both the sympathetic nervous system and the hypothalamus-pituitary-adrenal (HPA) axis, which complements the release stress factors such as serotonin, β-endorphin, corticotropin-releasing dopamine, glutamate, and norepinephrine, adrenocorticotropic hormones in the central nervous system (CNS), and the secretion of glucocorticoids in plasma [3,4,5]. Meanwhile, acute stress induces working memory impairment, anxiety, and depressive-like behaviors [6]. Evidence indicated that experimental animals repeatedly exposed to stress conditions can have altered cellular and hormonal immunity [7] as well as affected intestine microbiota [8]. Also, stress induces sympathetic stimulation and enhances metabolic rate, which can produce excess free radicals and lead to oxidative damage of lipids, proteins, and nucleic acids [9]. 

Certainly, exposure to acute stress like immobilization prompts the overexpression of pro-inflammatory cytokines in the CNS and triggers the transcription factor NF-κB-mediated pathways inducing expression of cyclooxygenase (COX-2), among other inflammatory mediators [10]. The evidence stated that acute stress is associated with the release of oxidative free radicals that facilitate the phosphorylation of mitogen-activated protein kinases (MAPK) cascade in the hippocampus of stress rats [11,12]. Immobilization is a suitable and easy method to cause physical stress, which leads to restricting mobility and aggression in an animal model [2] and is widely accepted for studying stress-induced alterations [13]. 

*Mentha arvensis* var is known as “corn mint” and is commonly used as Chinese medicine. Young stem tips can be used for food, and the whole plant has many medicinal properties, such as anti-inflammatory, antioxidant, and gastroprotective activities [14,15]. The major active compounds of MA extract such as hesperidin, rosmarinic acid, diosmin, didymin, buddleoside, acacetin and linarin have been determined by high-performance liquid chromatography (HPLC) analysis and reported for various biological effects [16,17]. Rosmarinic acid has been screened for antioxidant and anti-inflammatory potential [18,19]. Fermentation is an old technological process and diffusely used in various fields, including the food, drug, and cosmetic industries [20]. Fermentation sometimes can lessen toxicity [21], improve nutritional quality, [22] and increase digestive capacity [23] by enriching nutritional and functional properties [24]. It is well recognized that the traditional dietary pattern like fermentation can magnify nutrition quality [25]. However, *Lactobacillus rhamnosus*, *Enterococcus faecium*, and *Lactobacillus acidophilus* have been reported as the source of polyphenols [26] that increase antioxidant activity [27,28,29]. In this experiment, we first studied the protective effect of MA and particularly, MA that had been fermented with *Lactobacillus rhamnosus*, *Enterococcus faecium*, and *Lactobacillus acidophilus* (FMA). Based on the traditional uses and biological activities, our study investigates the anti-stress effect and underlying mechanism of MA and FMA in immobilized-stress in rats.

## 2. Results

### 2.1. Evaluation of Active Compound of MA and Content in MA and FMA 

**Figure 1:**
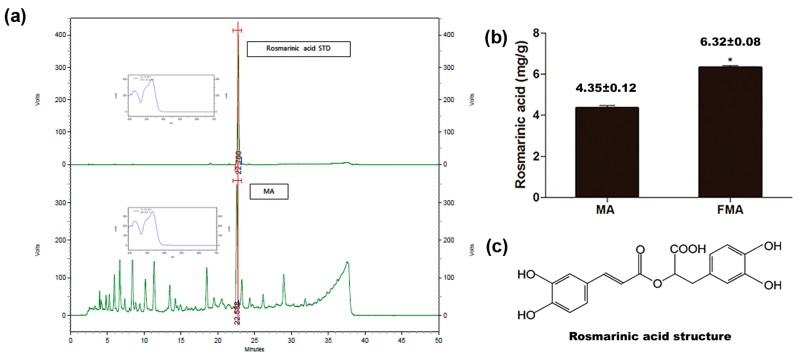
(**a**) High-performance liquid chromatography (HPLC) profile of rosmarinic acid standard and MA; (**b**) rosmarinic acid content in MA and FMA (* *p* < 0.05); (**c**) chemical structure of rosmarinic acid.

The active compound (rosmarinic acid) was identified in the water extract of MA (Figure 1a). The rosmarinic acid peak was observed at 22.6 min, and the pattern of the UV spectrum was also consistent, which confirmed that the material used in the experiment was MA. The rosmarinic acid content in MA and FMA was 4.35 ± 0.12 mg/g and 6.32 ± 0.08 mg/g, respectively (Figure 1b). The rosmarinic acid content in FMA was significantly higher as compared to MA. The chemical structure of rosmarinic acid is shown in Figure 1c.

### 2.2. Effects of MA and FMA Extract on Viability of RAW 264.7 Cells

**Figure 2:**
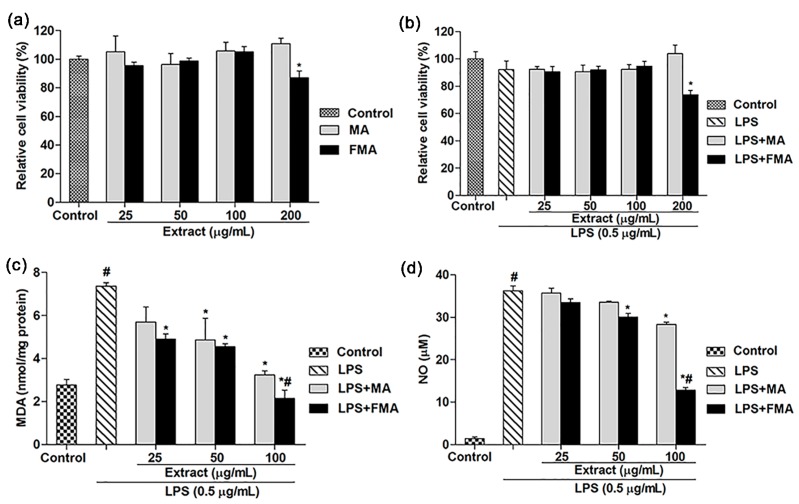
Effects of MA and FMA on (**a**) cytotoxicity and (**b**) cell viability in RAW 264.7 cells were determined by MTT assay; (**c**) MDA and (**d**) NO in RAW 264.7 cells were determined by TBARS and Griess assays. Cells were pretreated with various concentration of MA and FMA extract (25, 50, and 100 μg/mL) for 1 h, followed by co-treatment with LPS (0.5 μg/mL) for another 24 h. # *p <* 0.05 when compared with control and * *p <* 0.05 when compared with LPS. Data are expressed as mean ± SEM of three independent experiments.

The protective effects of MA and FMA were assessed using RAW 264.7 cells on LPS-induced inflammation by 3-(4,5-dimethylthiazol-2-yl)-2,5-diphenyltetrazolium bromide (MTT) assay. To measure the nontoxic concentration of MA and FMA, RAW 264.7 cells were treated with various concentration of MA and FMA (25, 50, 100, and 200 μg/mL) for 24 h. A marked (*p <* 0.05) decrease of cell viability was found at FMA (200 μg/mL) as compared to the control group (Figure 2a). Furthermore, the cell viability did not significantly decrease by co-treatment with MA and FMA extract (25, 50, and 100 μg/mL) for 24 h (Figure 2b).

### 2.3. Effect of MA and FMA on MDA and NO Release in RAW 264.7 Cells

RAW264.7 cells were treated with 0.5 μg/mL of LPS for 24 h which caused a marked (*p <* 0.05) increase in the intracellular MDA level compared with the control group, whereas pre-incubation of cells with MA (50 and 100 μg/mL) and FMA (25, 50, and 100 μg/mL) significantly (*p <* 0.05) decreased the MDA level (Figure 2c). Moreover, at the concentration of 100 μg/mL, FMA extract had more ability (*p* < 0.05) in decreasing the MDA level than MA extract. In addition, as shown in Figure 2d, NO content significantly (*p <* 0.05) increased after treatment with 0.5 μg/mL of LPS for 24 h. Furthermore, pre-incubation with MA (100 μg/mL) and FMA (50 and 100 μg/mL) before LPS exposure significantly (*p <* 0.05) decreased the NO concentration compared to LPS treatment alone. 

### 2.4. Effect of MA and FMA on Body Weight and Food Intake

**Figure 3:**
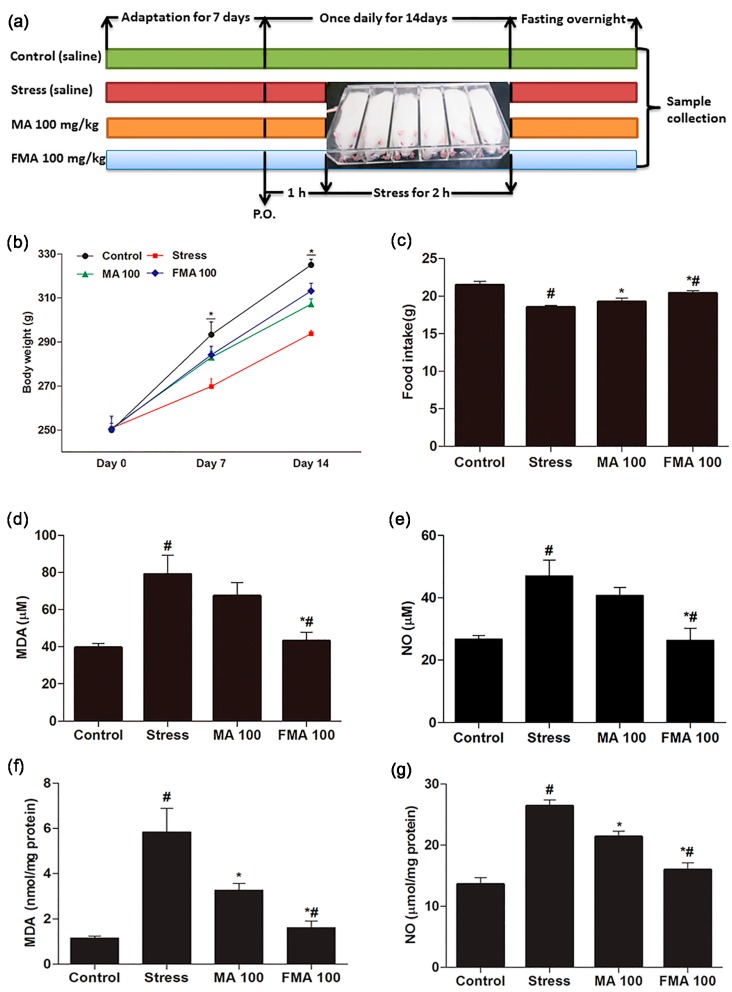
Effect of MA and FMA extract on body weight, food intake, and oxidative stress markers in immobilization stress-induced rats. (**a**) Rats were orally administered 100 mg/kg MA (MA 100) and 100 mg/kg FMA (FMA 100) extract 1 h prior to immobilization; (**b**) body weight and (**c**) food intake was measured during the experimental period; Serum concentration of (**d**) MDA; (**e**) NO and hippocampus concentration of (**f**) MDA; (**g**) NO were assayed. Data are expressed as mean ± standard error (SEM). # *p <* 0.05 compared with the control and tress group; MA 100 and FMA 100 group, * *p <* 0.05 compared with the stress group and MA 100 and FMA 100 groups.

Immobilization stress significantly decreased accumulated body weight gain compared to the control group. Pretreatment with MA and FMA extract significantly (*p <* 0.05) improved the body weight of rats up to 14 days as compared with the immobilized rats (Figure 3b). Similarly, the food intake showed a significant (*p <* 0.05) decrease in the stressed group compared to control group, whereas rats pretreated with the MA and FMA extract had significantly (*p <* 0.05) improved food intake during the experimental period (Figure 3c). These results revealed that both MA and FMA extracts improved the body weight and food intake in immobilized rats. FMA had a better effect (*p <* 0.05) on body weight and food intake than MA. 

### 2.5. Effect of MA and FMA on MDA and NO Production in Immobilized Rats Serum and Hippocampus

The concentration of serum and hippocampus MDA was notably (*p <* 0.05) increased in immobilized rats. Pretreatment with MA and FMA extract markedly (*p <* 0.05) mitigated the serum and hippocampus level of MDA as compared with the immobilization stress group. This reduction was particularly significant in the FMA extract (100 mg/kg) treatment group (Figure 3d,f). On the other hand, to demonstrate whether immobilization stress can induce inflammation in rats, we measured the serum and hippocampus concentration of NO, which was significantly (*p <* 0.05) increased in the stress group as compared to the control group. Pretreatment with MA and FMA extracts ameliorated the serum and hippocampus NO levels (*p <* 0.05) in immobilized rats and predominantly FMA extract significantly (*p <* 0.05) decreased the serum and hippocampus NO levels (Figure 3e,g)

### 2.6. Effect of MA and FMA on Serum Corticosterone, β-Endorphin, and Serotonin in Immobilized Rats

**Figure 4:**
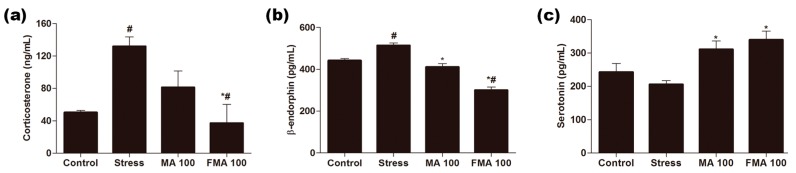
Effect of MA and fermented MA (FMA) extract on serum (**a**) corticosterone; (**b**) β-endorphin; and (**c**) serotonin in immobilization stress-induced rats. Rats were administered 100 mg/kg MA (MA 100) and 100 mg/kg FMA (FMA 100) extract 1 h prior to immobilization. Serum corticosterone, β-endorphin, and serotonin were assayed by enzyme-linked immunosorbent assay (ELISA). Data are expressed as mean ± standard error (SEM). # *p <* 0.05 compared with the control and stress group; MA 100 and FMA 100 group, * *p <* 0.05 compared with the stress group and MA 100 and FMA 100 groups.

Immobilization stress markedly (*p <* 0.05) increased serum level of corticosterone and β-endorphin compared with the control group. Pretreatment with MA and FMA extract in stress-induced rats inhibited the induction of serum concentration of corticosterone and β-endorphin (Figure 4a,b). Mostly, FMA extract revealed an anti-stress effect more potently on decreasing (*p <* 0.05) corticosterone and β-endorphin than MA extract. Besides, in the immobilized rats, the serum serotonin level slightly declined compared with the normal control group. Pretreatment with MA and FMA extract showed a marked (*p <* 0.05) increase in serotonin level compared to the stress group (Figure 4c). Individually, FMA extract significantly (*p <* 0.05) regulated the stress-related physiological indicators better than MA extract.

### 2.7. Effect of MA and FMA on Histology of the Duodenum

**Figure 5:**
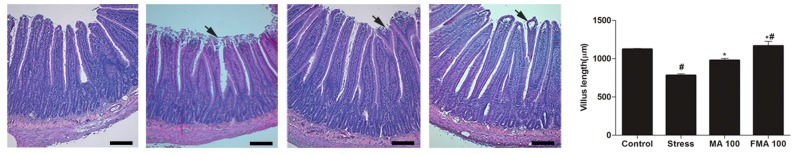
Effect of MA and FMA extracts on duodenum histology and villus length in immobilization stress-induced rats. Rats were administered 100 mg/kg MA (MA 100) and 100 mg/kg FMA (FMA 100) extract 1 h prior to immobilization. Duodenum histology was markedly improved by MA and FMA co-treatment groups as compared to the stress group. The black arrow indicates a damaged mucosal layer of the duodenum. Scale bar 200 μM. Data are expressed as mean ± standard error (SEM). # *p <* 0.05 compared with the control and stress group; MA 100 and FMA 100 group, * *p <* 0.05 compared with the stress group and MA 100 and FMA 100 groups.

We investigated the anti-stress effects of MA and FMA extract on immobilized stress-induced oxidative stress in the intestinal mucosa. We found out that the mucosa of the duodenum was slightly damaged in stressed rats compared to the control group. Pretreatment with MA and FMA extracts notably improved the histology of the duodenum (Figure 5). Moreover, a significant (*p <* 0.05) increase of villus length was observed in MA and FMA-treated rats compared to the stress group. Particularly, when the rats ingested 100 mg/kg FMA extract, the villus length was a little longer than in the MA 100 group in immobilized rats (Figure 5).

### 2.8. Effect of MA and FMA on MAPK/COX-2 Signaling Pathways

**Figure 6:**
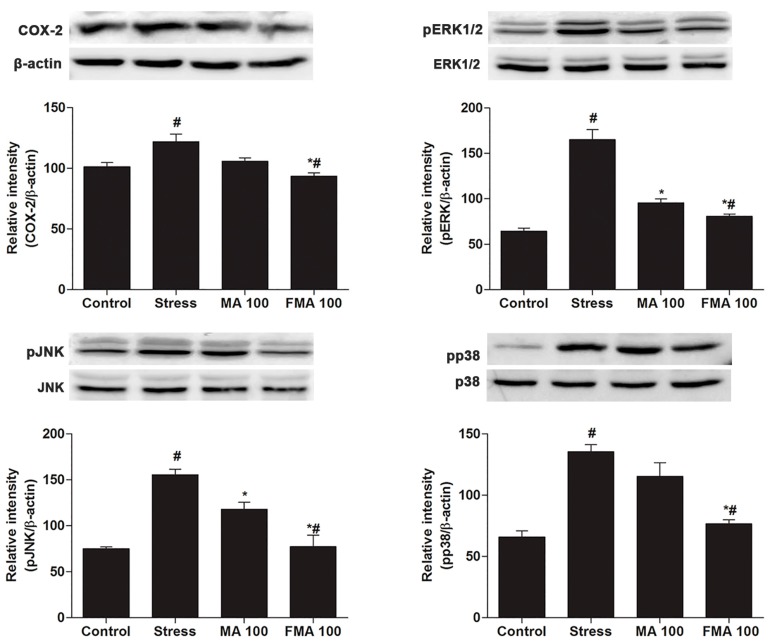
Effect of MA and FMA extract on the mitogen activated protein kinase (MAPK) cascade phosphorylation (ERK1/2, JNK and p38) and cyclooxygenase-2 (COX-2) expression in hippocampus tissue of immobilization stress-induced rats. The relative band intensity was measured as compared with β-actin and total MAPK. Immobilized stress-induced the marked phosphorylation of MAPK cascade and COX-2 expression; in contrast, treatment with the MA and FMA reduced the phosphorylation of MAPK cascade and COX-2 expression, respectively. # *p <* 0.05 compared with the control and stress group; MA 100 and FMA 100 group, * *p <* 0.05 compared with the stress group and MA 100 and FMA 100 groups.

To evaluate the possible molecular mechanisms of the anti-stress role of MA and FMA extract, we evaluated the protein expression related to anti-stress. Immobilized-induced phosphorylation of MAPK family proteins such as extracellular signal-regulated kinases (ERK1/2), c-Jun N-terminal kinase (JNK), and p38 levels in the hippocampus was notably (*p <* 0.05) attenuated by MA and FMA treatment (Figure 6). For the meantime, the COX-2 expression in hippocampus tissues was markedly (*p <* 0.05) blocked by MA and FMA (Figure 6). Furthermore, FMA shows a better ability on suppressing the MAPK family proteins and COX-2 level.

## 3. Discussion

Immobilization stress is a state that threatens homeostasis and causes a variety of psychological, physiological, and pathological disorders [30]. These contribute to stress responses, including neuronal, endocrine, and immune reactions, which could lead to interference with host defenses [31]. 

*Mentha arvensis* has an enriched and abundant natural phenolics and flavonoids content, its antioxidant and anti-inflammatory properties may be observed due to the presence of various chemically active components. This indicates that the crude drugs may have an antioxidant and anti-inflammatory effect due to their polyphenolic property. Among them, rosmarinic acid is a water-soluble polyphenol, which can be isolated from many herbs. Previous studies have shown that rosmarinic acid possesses various biological properties, including anti-oxidative, anti-inflammatory and anticancer properties [19,32,33]. Before the study, we found rosmarinic acid by HPLC analysis, and then measured its content in MA and FMA extracts. It was discovered that rosmarinic acid in FMA extract was much higher than MA extract. MA and FMA extract co-treatment did not significantly affect the cell viability in RAW 264.7 cells for 24 h. MDA and NO are the mediators of the regulation of cell functions and inflammatory biomarkers [34]. Previous studies have shown that rosmarinic acid decreased the oxidative stress-mediated NO production in RAW 264.7 cells [35]. Oxidative stress is recognized to play a significant role in the pathogenesis of immobilized stress injury [36]. Rats exposed to acute immobilization stress have high concentrations of oxidative free radicals in their body system. The free radicals initiate lipid peroxidation [37] and alter the prooxidant-antioxidant balance, and increase the development of various pathological states, such as neuronal damage, disruption of energy pathways, and induction of signaling events in apoptotic cell death [36]. *Lactobacillus rhamnosus* [38], *Enterococcus faecium* [27] and *Lactobacillus acidophilus* [39] have been reported to improve antioxidant capacity, perhaps because of microbial metabolic activity during fermentation [40]. In the present study, pretreatment with MA and FMA extracts ameliorated MDA and NO synthesis in RAW 264.7 cells and immobilized-stress rats. Both extracts demonstrated their antioxidant property by reducing production of MDA and NO levels. Our result was supported by an earlier study [37]. 

In this study, experimental rats were fed with commercial standard food. Compared with a control group, immobilization stress significantly decreased body weight gain and accumulated feed intake in rats, perhaps because of their inability to feed during immobilization. Our finding correlated with other studies which showed that immobilization stress reduces weight gain and food consumption in rats [41]. A slight reduction in body weight gain induced by stress is regulated and is partially affected by anorexia [42]. However, pretreatment with MA and FMA extracts ameliorated the body weight and food intake in immobilized stress rats. 

The oxidative, endocrine and nervous systems are involved in stress response in the body [43]. Corticosterone is the richest glucocorticoid (GC) in rats [44] and has been considered as a valuable serum marker of the stress condition in rodents [45]. Increased serum corticosterone level is consistent with the suggestion that physiological responses to repeated stress are associated with the activation of the HPA axis [46]. As with these studies, we observed a significant increase in serum corticosterone after immobilization stress [47]. However, both MA and FMA extract administration significantly reduced the increase in corticosterone levels. Previous data revealed that rosmarinic acid notably reduced the serum corticosterone level in tail suspension test (TST)-induced stress [48]. β-endorphin, an endogenous opioid, is produced in the pituitary gland; it is released into the circulating blood to act as a relaxing factor against stress [49]. β-endorphin adjusts pain perception in both the central nervous system and the peripheral nervous system [50]. In our study, serum β-endorphin level was significantly increased in the immobilization-stress group. The result was like that of previous studies [51,52,53]. However, pretreatment with MA and FMA extract gradually reduced the concentration of β-endorphin as compared to the stress group. Moreover, serotonin is a chemical produced by nerve cells and sends signals between them; 90% of serotonin is found in the digestive system and blood platelets and is essential for regulating mood and emotion. Abnormal serotonin plays a key role in the stress response and the mechanism of antidepressant action [54]. Exposure to stress causes anxiety disorders at the early-time period and then induces depression, which is related at least partially to the decreased level of serotonin [1]. In our experiment, the level of serotonin was notably decreased after the rat exposure to immobilization. Our result was similar to the earlier study [55]. Pretreatment with MA and FMA significantly improved the serotonin level as compared to the stress group. Imbalance of serotonin level causes depression and anxiety disorders. As is widely revealed by the fact that most antidepressants increase the extracellular serotonin level [56]. 

Several studies indicate that different types of physical (immobilization) and psychological stress affect several components of intestinal mucosal function [57]. Lipid peroxidation is the result of an oxidative free-radical reaction against cell membrane and produces a marked amount of MDA, which could lead to intestinal mucosal damage [58]. Studies have shown that rosmarinic acid ameliorates oxidative stress-mediated lipid peroxidation in rats [59]. In our study, the mucosal layer was damaged and the villus length was significantly reduced after immobilization stress, leading to a diminution in absorption [60]. Different types of psychological and physical stressors have a significant influence on several components of intestinal mucosal function, which is thought to contribute to symptoms of chronic inflammatory diseases and functional disorders of the gastrointestinal tract [61]. Pretreatment with MA and FMA extract recovered the mucosal structure and villus length. Fermentation has been reported to increase production of bioactive compounds and provide health benefits beyond basic nutrition and health promotion properties by producing various biomolecules [62]. Furthermore, the FMA extract showed a stronger ability in promoting the mucosal structure and length of villi than MA extract.

Oxidative stress is correlated with immobilized stress. In physiological stress, ROS-mediated changes in lipids, protein, and DNA could distress the CNS functions [63]. Excessive oxidative stress produces oxidative free radicals such as ROS which stimulate the MAPK cascade phosphorylation [64]. Oxidative modification of macromolecules can stimulate signaling pathways, membrane restoration, and gene transcription. Certain inflammatory mediators such as COX-2 are involved in the physiological dysfunction of brain tissue [65]. In this investigation, MAPK cascade phosphorylation and COX-2 expression were markedly increased in the hippocampus. It seems to correlate with the MAPK/COX-2 activity in immobilized-stress. However, MA and FMA treatment gradually control this event which has been supported by an earlier study [65]. In relation to MA and FAM, rosmarinic acid-inhibited oxidative-stress-induced MAPK phosphorylation and COX-2 expression in a rat model [66]. MA and FMA modified the stress-induced MAPK/COX-2 activity in hippocampus and, thereby, mitigated the immobilized-stress in rats.

## 4. Materials and Methods

### 4.1. Analytical Reagents and Chemicals

The highest analytical grades of all chemicals were used. Lipopolysaccharide (LPS), penicillin/streptomycin, 3-(4,5-dimethylthiazol-2-yl)-2,5-diphenyltetrazolium bromide (MTT), hematoxylin, eosin, rosmarinic acid, and protease inhibitor were purchased from Sigma-Aldrich (St. Louis, MO, USA). Trypsin–Ethylenediaminetetraacetic acid (EDTA) and fetal bovine serum (FBS) were collected from GE Healthcare (Chicago, IL, USA). Dulbecco’s Modified Eagle’s Medium (DMEM), and other cell-culture reagents were obtained from Gibco (Carlsbad, CA, USA). T-per buffer and BCA protein assay kit was bought from Thermo Scientific (Waltham, MA, USA), Primary antibodies (phospho-ERK1/2, phospho-JNK phospho-p38, and COX-2) and β-actin antibody were provided by Cell Signaling (Danvers, MA, USA). The secondary antibody (goat anti-rabbit IgG-HRP) was obtained from Santa Cruz Biotechnology (Santa Cruz, CA, USA). Enzyme-linked immunosorbent assay (ELISA) kit (corticosterone) was purchased from R&D Systems (Minneapolis, MN, USA), and β-endorphin and serotonin ELISA kits were purchased from Elab Science (Wuhan, China). TBARS kit was supplied by Cayman (Ann Arbor, MI, USA) and Griess assay kit was obtained from Thermo Scientific (Waltham, MA, USA). Isoflurane was obtained from JW Pharmaceutical (Seoul, Korea). 

### 4.2. Preparation of Mentha Arvensis var Extract

*Mentha arvensis* var (MA) was purchased from Omnihub Co. (Daegu, Korea). MA was dried and pulverized using a freeze dryer (FDA5508, ilShinbiobase. Co., Ltd., Dongducheon, Korea). Three strains of *Lactobacillus rhamnosus* (L3 KCTC18485P), *Enterococcus faecium* (L54 KCTC18486P), and *Lactobacillus acidophilus* (L120 KCTC18487P) were isolated from the feces of elderly people. 600 mL of water was added to 30 g of MA powder, and the mixture was stirred at 100 °C for 9 h. The extract was filtered using a filter paper (ADVANTEC No. 2, Adventec Toyo Kaisha, Tokyo, Japan). The filtrate was concentrated on a rotary evaporator (EYELA, N-N series, Tokyo, Japan) and lyophilized. For the preparation of the fermented MA (FMA), the MA extract was adjusted to 10° Brix and sterilized at 121 °C for 15 min. 5% mixture of L3, L54, and L120 strains (1:1:1, 1.0 × 10^6^ CFU/mL) were used. MA extract was fermented at 37 °C for 48 h. After fermentation, the supernatant was collected by a centrifuge (10,000 rpm, 5 min, 4 °C) (VS-5000N, Vision Science Co., Daegu, Korea) and sterilized at 121 °C for 15 min. The dried extract was kept at −20 °C. The study was conducted using a single batch of plant extract to avoid batch-to-batch differences and maximize the product constancy.

### 4.3. HPLC Analysis and Measurement of the Content of the Rosmarinic Acid in MA and FMA Extract

The phytochemical characteristics of *Mentha arvensis* and the standard compound rosmarinic acid were identified by HPLC analysis (Nanospace SI-2, Shiseido, Tokyo, Japan). The column is Capcellpak MG II (Φ4.6 × 250 mm, 5 μm, Shiseido); PDA detector (330 nm) was used. The column temperature was 40 °C. The injection volume was 10 µL and the flow rate was 1.0 mL/min. To determine the content of rosmarinic acid in MA and FMA we measure the peak area in a specific time period during HPLC analysis. The content of rosmarinic acid in MA and FMA was presented in Figure 1b.

### 4.4. Cell Culture and Assessment of Cell Viability

RAW 246.7 cell line was grown in DMEM with 10% FBS and 1% penicillin, streptomycin, and incubated at 37 °C in 5 % CO_2_. Cell viability was measured by MTT assay [67]. RAW 246.7 cells were seeded (1 × 10^4^ cells/well in 96-well plates) and cultured in a 37 °C incubator overnight. To evaluate the cytotoxicity of the MA and FMA extracts, cells were treated with different concentrations of MA and FMA extracts (25, 50, 100, and 200 μg/mL) for 24 h, respectively. Briefly, for measuring the cell viability, cells were separately pretreated for 1 h with different concentration of MA and FMA extracts (25, 50, 100, and 200 μg/mL) and then co-incubated with LPS (0.5 μg/mL) for an additional 24 h. The medium was replaced with 0.5 mg/mL of the MTT working solution and incubated for 2 h. The blue formazan crystals were solubilized with DMSO. Optical density was measured at 570 nm absorbance by a tunable versa max microplate reader (Molecular Devices, Sunnyvale, CA, USA). 

### 4.5. Measurement of MDA and NO Content in RAW 264.7 Cells

MDA and NO concentration were measured using TBARS kit and Griess reagent kit according to the manufacturer’s instructions. Optical density was measured at 535nm (MDA) and 548 nm (NO) absorbance by a tunable versa max microplate reader (Molecular Devices, Sunnyvale, CA, USA). 

### 4.6. Animal Management and Experimental Design

Sprague-Dawley rats (7 weeks-old, 220 ± 10g) were acclimated for 7 days before the experiment and maintained in accordance with the animal welfare regulations of the Institutional Animal Care and Use Committee (IACUC; CBNU 2016-68), Chonbuk National University Laboratory Animal Centre, South Korea followed by the NIH guide for the care and use of laboratory animals. All rats were kept at an optimal temperature (25 ± 1 °C), and humidity (50 ± 10%) and photoperiod cycle (12 h light and 12 h dark) were maintained during the experimental period. The rats were fed *ad libitum* with standard food and distilled water. For inducing immobilization stress in rats, we followed an assigned method with minor modification [68]. A total of 32 rats were randomly divided into 4 groups: (1) Normal control; (2) Stress (immobilization); (3) MA 100 (stress + MA 100 mg/kg); and (4) FMA 100 (stress + FMA 100 mg/kg). Each extract was orally administered to rats 1 h prior to immobilization. Rats were immobilized for 2 h per day for 14 days using a restraining chamber [69] (Figure 3a). After the experiment, the rats were fasted overnight, anesthetized with Isoflurane, and then euthanized by cervical dislocation. Blood samples were collected directly from the cardiac puncture, and serum was separated. Tissue samples (duodenum) were collected immediately and fixed in 10% neutral buffered formalin (NBF) subjected to H&E staining. 

### 4.7. Measurement of Body Weight and Food Intake

Rats were weighed on day 0, day 7, and day 14 prior to immobilization. Throughout the experimental period, we also monitored the food intake every day.

### 4.8. Measurement of MDA and NO Content in Rat Serum and Hippocampus

Blood samples were kept at room temperature for 30 min and centrifuged at 4 °C for 15 min at 3000 rpm. Serum was collected and stored at −80 °C. The MDA and NO concentration in serum and hippocampus were measured using a TBARS kit and Griess reagent kit according to the manufacturers’ instructions.

### 4.9. ELISA Assay for Corticosterone, β-endorphin, and Serotonin

Serum concentration of corticosterone, β-endorphin, and serotonin was assayed by ELISA. The tests were performed according to the manufacturers’ specifications.

### 4.10. Histological Analysis

For histological analysis, we followed a given method [70]. Briefly, the duodenum was immediately fixed in 10% NBF; 5 μm sections were made and subjected to H&E staining. Digital images were obtained using a Leica DM2500 microscope (Leica Microsystems, Wetzlar, Germany) at a fixed 100 × magnification. The villus length was measured using image measurement software (v 22.1., iSolution DTM, Vancouver, BC, Canada).

### 4.11. Western Blot Analysis

Brain hippocampus was harvested and washed twice with ice-cold PBS. Tissues were lysed by the lysis buffer; tissue protein extraction reagent (T-PER), phenylmethanesulfonyl fluoride (PMSF), sodium orthovanadate (Na_3_VO_4_), and protease inhibitor cocktail. The total concentration of protein of lysate tissues was measured with a bicinchoninic acid (BCA) protein assay protein kit. An equal amount of protein was separated by 12% sodium dodecyl sulfate-polyacrylamide gel electrophoresis (SDS-PAGE) and transferred to a nitrocellulose membrane. The membrane was incubated with blocking serum; 5% bovine serum albumin (BSA) in Tris-buffered saline with tween twenty (TBST) for 2 h at room temperature and by primary antibodies for overnight at 4 °C. Then, the blot was washed and incubated with secondary antibodies for 2 h. Bands were detected using an enhanced chemiluminescence (ECL) detection kit, and bands images were taken by a LAS-400 image system, (GE Healthcare, Little Chalfont, UK); β-actin was used as the reference antibody.

### 4.12. Statistical Analysis

Data were analyzed with Graph Pad Prism 5.0 (GraphPad Software, Inc., 7825 Fay Avenue, Suite 230, La Jolla, CA, USA) and expressed as mean ± standard error (SEM). Groups were compared using one-way and two-way analysis of variance (ANOVA), followed by Bonferroni’s multiple comparison tests and student’s *t*-test. For comparison of groups, we used two-way ANOVA for body weight and one-way ANOVA for other data analysis. Student’s *t*-test was used to compare the MA 100 and FMA 100 groups. The minimum statistical significance was considered to be *p <* 0.05 for all analyses.

## 5. Conclusions

Our study revealed that MA and particularly, FMA extract had a potent anti-stress effect on immobilized stress that could operate by attenuating oxidative stress, regulating the stress-related hormones (corticosterone, β-endorphin, and serotonin) and MAPK/COX-2 signaling pathways. These finding suggested that MA and FMA are natural phytomedicines that could be used for anti-stress-induced oxidative disorders in humans.
